# Improving Major Depressive Episode Assessment: A New Tool Developed by Formal Psychological Assessment

**DOI:** 10.3389/fpsyg.2017.00214

**Published:** 2017-02-17

**Authors:** Francesca Serra, Andrea Spoto, Marta Ghisi, Giulio Vidotto

**Affiliations:** Department of General Psychology, University of PaduaPadova, Italy

**Keywords:** major depressive episode, questionnaire, formal psychological assessment, symptomatology, psychopathology, quantitative-qualitative evaluation

## Abstract

**Aim:** Major depressive episode (MDE) can manifest with different features. Discriminating between different types of MDEs is crucial for proper treatment. The aim of this study is to propose a new tool for MDE assessment in bipolar disorder (BD) or major depressive disorder (MDD) to overcome some limitations of current rating scales. The proposed tool investigates all of the clinical features of different MDEs and gives qualitative information, differentiating patients with the same score but different symptoms and psychopathology severity. To achieve this purpose authors used a new methodology called Formal Psychological Assessment (FPA). FPA allows creating relations between the items of an assessment tool, and the set of diagnostic criteria of a given clinical disorder. In the application at hand, given the capability to analyze all clinical features, FPA appears a useful way to highlight and differentiate between inhibited and agitated depressive symptoms.

**Method:** The new tool contains 41 items constructed through 23 clinical criteria from the DSM-5 and literature symptoms. In line with FPA, starting from a set of items and a set of clinical criteria, a Boolean matrix was built assigning to each item its own set of clinical criteria. The participants include 265 in the control group and 38 patients with MDE (diagnosed with MDD or BD) who answered the QuEDS. After 1 month, 63 participants performed the test again and 113 took the Depression-Anxiety-Stress Scale to analyze convergent—divergent validity.

**Results:** The scale showed adequate reliability and validity. A hierarchical confirmatory factor analysis highlighted the presence of three sub factors (affective, somatic, and cognitive) and one high-order factor (depression).

**Conclusions:** The new tool is potentially able to inform clinicians about the patients' most likely diagnostic configuration. Indeed, the *clinical state* of a patient consists of the subset of items he/she answered affirmatively, along with his/her subset of specific symptoms. Qualitative information is fundamental from a clinical perspective, allowing for the analysis and treatment of each patient according to his/her symptoms in an effective way.

## Introduction

Clinical evaluation and treatment may have different aims; in fact, psychologists and psychiatrists face many forms of suffering and discomfort. The case formulation is obtained by collecting all necessary data, which allows clinicians to reconstruct the mechanisms and processes underlying the disorders presented, agree on treatment goals, and to identify the most appropriate therapy in an effective way (Grossberg, 1964; Groth-Marnat, 2009; Bokhari and Hubert, 2015).

Even after following the relevant steps of assessment research, some critical issues still exist. Specifically, the tools for evaluating mood disorders, particularly those involving major depressive episodes (MDEs) ones, show some application limits. Some studies highlighted critical issues regarding self-report depression tools and this is crucial for assessment and treatment (Baldessarini et al., 2010; Hyman, 2014).

Pettersson et al. (2015) conducted a systematic review on evaluating depression, which revealed that only the Structured Clinical Interview for DSM-IV Axis I Disorders (SCID-I; First et al., 1995), the Mini-International Neuropsychiatric Interview (MINI; Sheehan et al., 1998), and the Patient Health Questionnaire (PHQ-9; Manea et al., 2012) fulfilled the minimum criteria for sensitivity and specificity. Out of these three tools, only the PHQ-9 is a self-report measure that can be used for screening, diagnosis, monitoring, and measuring the severity of depression.

In a critical study, Balsamo and Saggino (2007) introduced the psychometric strengths and weaknesses of the most important self-assessment measures of depression. The purpose was to avoid confusion in clinical practice. Specifically, the study explored the psychometric properties of six self-report measures of depression: the Beck Depression Inventory-II (BDI-II; Beck et al., 1988); the Center for Epidemiological Studies Depression Scale (CES-D; Radloff, 1977); the Zung Self-Rating Depression Scale (Sakamoto et al., 1998); the Clinical Depression Questionnaire (CDQ; Krug and Laughlin, 1976); the Questionnaire for Depression (QD), included in the Cognitive Behavioral Assessment 2.0 battery (CBA 2.0; Sanavio et al., 1986); and the D scale of the Minnesota Multiphasic Personality Inventory (MMPI; Hathaway and McKinley, 1942). Balsamo and Saggino (2007) showed that patients overestimate symptoms, perhaps because of their mental condition; another observed limitation was that each scale reflects the authors' theories, which were constructed to measure different aspects of the same construct.

A study of seven widely used self-report measures on depression (Serra et al., 2015) highlighted the weaknesses of these tools in relation to their ability to investigate all of the diagnostic criteria for MDE described by the DSM-5 and a large amount of literature. Attention was focused on seven self-assessment questionnaires: the BDI-II (Beck et al., 1996), the SDS (Zung et al., 1965), the Rome Depression Inventory (RDI; Pancheri and Carilli, 1982), the Plutchik–Van Praag self-report depression scale (PVP; Plutchik and Van Praag, 1987), the Carroll Rating Scale (CRS; Carroll et al., 1981), the Self-Assessment Scale for Depression (SAD; Cassano and Castrogiovanni, 1982), and finally, the Center for Epidemiological Studies Depression scale (CES-D; Eaton et al., 2004; Shean and Baldwin, 2012). Different tools measure different aspects of depression (cognitive, emotional, physiological, etc.); therefore, it is important to know which aspects a depression scale is able to identify. This study reported that none of the seven tools investigates the integration of clinical criteria by itself.

Dobson (1985) found poor discriminant validity between depression and anxiety, highlighting the high correlation (around 0.61) among the different tools for measuring depression and anxiety.

Gibbons et al. (1985) emphasized that the traditional method of scoring can be “wrong” because it is based on assumptions that may be false: it gives equal weight to each item, assuming that each item or symptom of a clinical scale represents an equal level of psychiatric severity.

Moreover, as many authors have underlined, self-evaluating questionnaires allow for a systematic and quick collection of a large amount of information and the avoidance of patient embarrassment; on the other hand, they redundantly (non-adaptively) investigate constructs and provide only a quantitative numeric score that does not systematically account for qualitative information (Shapiro, 1951; Wright and Feinstein, 1982; Fava et al., 2004; Spoto et al., 2013; Bottesi et al., 2015b).

Based on the assumptions of the studies described above, it is important to consider all of the limits that self-report tools have, by taking into account the possible overestimation of symptoms by patients (Faravelli et al., 1986; Balsamo and Saggino, 2007), the high comorbidity between depression and anxiety (Dobson, 1985), and the inability of MDE self-report measures to enclose the whole set of depressive symptoms, whether agitated or inhibited (Koukopoulos and Koukopoulos, 1999; Serra et al., 2015), in the construction of the item (including the PHQ-9-Sensitive estimated in Petterson's review). Finally, it is also relevant to remember not to take into account only the patient's cutoff scores (Gibbons et al., 1985; Fava et al., 2004; Bottesi et al., 2015b). As a consequence of the last statement, even overtaking or not overtaking the cutoff may not always be so important. In fact, the cutoff provides only a quantitative score, but if two patients have the same score, it does not mean that they have equal symptomatology (one could be much more serious than the other, since he/she responded positively to more severe symptoms).

The purpose of this study is to create a new tool for MDE evaluation to overcome the difficulties of the MDE tools described above. The present study aims to construct an adaptive-qualitative tool that investigates all MDE clinical features and provides qualitative information (and not just a score) to differentiate patients with the same score but different symptoms as well as differing severities of psychopathology. From a conceptual point of view, depression is a strong and united construct; some authors, including Beck, attributed more importance to the cognitive dimension of depression (Rainone and Mancini, 2007) without neglecting the somatic-affective dimension. Many authors identified three dimensions in the depression construct: cognitive, somatic, and affective (O'Hara et al., 1984; Roca et al., 1996; Dinger et al., 2015). A hierarchical model having three subdimensions (namely: cognitive, affective and somatic) and one second order factor (depression) could be the factorial solution that is most likely to represent the structure of the investigated construct; in fact, this model would have the advantage of offering an interesting explanation, from the clinical perspective.

One possibility to overcome some of the assessment limitations and therefore to build up this new tool is a new methodology called Formal Psychological Assessment (FPA; Spoto et al., 2010, 2013; Bottesi et al., 2015b; Serra et al., 2015). FPA tries to maximize the advantages of both self-report tests and semi-structured interviews. The FPA was born as an extension to the CBA-2.0 (Bertolotti et al., 1990) and developed in an original way from the conjunction of two mathematical psychology theories and a consequent innovative clinical application: Knowledge Spaces Theory (KST; Doignon and Falmagne, 1985, 1999; Falmagne and Doignon, 2011) and Formal Concept Analysis (FCA; Wille, 1982; Ganter and Wille, 1999). FPA allows the detection and fruitful use of relations among a set of items (*objects*) from any clinical tool and a particular set of diagnostic criteria (*attributes*) for a given clinical disorder. Below some key concepts of this approach are described.

In FPA, each item included in a clinical self-report questionnaire (or interview) is defined as an *object*. Each item can be described on the basis of a set of elements (symptoms) referring to a given theoretical framework. These symptoms come from the decomposition of the diagnostic criteria from the DSM-5 (American Psychiatric Association, 2013) for a given disorder, or from additional clinical criteria derived from the literature with respect to a particular clinical disorder. The symptoms are named *attributes*, and each object can be related to the set of attributes it endorses. Each item may investigate one or more attributes (symptoms), and each attribute can characterize one or more items. For example, a hypothetical item “*I have sleep problems”* investigates the attribute *insomnia or iper-somnia* (DSM-5, major depressive episode). Starting from a set of items and a set of attributes, a *Boolean matrix* can be built that assigns to each item its own set of clinical criteria (*attribute*). The items are placed in the rows of the matrix, and the attributes are placed in the columns. Every time an item investigates a specific attribute, the corresponding cell of the matrix will contain “1”; otherwise, the cell will contain “0” (Spoto et al., 2013; Bottesi et al., 2015b; Serra et al., 2015). In FPA, this matrix represents the *clinical context*. The entire set of item of a clinical tool is the *domain* of the clinical context. A patient's *clinical state* consists of the subset of items he/she answered affirmatively. Thus, even if two patients respond affirmatively to the same number of items (i.e., they obtain the same score in the questionnaire), the representation of their two states in terms of attributes will be different, if the items they answered affirmatively are also different. The *clinical structure* represents the relationship among the items of the domain. The implications of the items form prerequisite relations. The prerequisite is an item that contains the same attribute “a” of another item that contains the same attribute “a” and one or more (for example “b”). In total, the second item will contain “a” and “b.” The first item is necessary to get a positive answer to the second item. The prerequisite relation among the items obtained from the matrix through the formal mathematical passages can lead to the development of adaptive and qualitative tools as well as quantitative tools (Spoto et al., 2010; Donadello et al., 2016).

The paper is organized as follows: in the next section is described the tool construction, including the choice of the clinical criteria and the construction of the items. In that section are provided also the methodological details about the sample, the procedure and the data analysis. In Section Results, results of the psychometric study regarding the reliability and validity of the new tool are displayed, together with two clinical cases examples highlighting the specific improvement in the collected information allowed by the new tool. Future perspectives, limitations and strengths of the present study are finally discussed in Section Discussion.

## Materials and methods

### Tool construction

Three important steps were achieved in constructing the new tool:

1. Various features and symptoms (clinical criteria) of MDEs were analyzed and categorized as attributes. As described in the DSM-5 (American Psychiatric Association, 2013), there are different types of MDEs; in particular, a MDE may be reactive (due to bereavement or trauma) or have a biological basis, and it can be part of MDD or bipolar disorder (BD, Type I or II), with symptoms more agitated or more inhibited.
2. The items were constructed on the basis of one or more chosen clinical criteria (attributes).
3. In line with the FPA methodology, the matrix was obtained to analyze all of the relationships among items (objects) and diagnostic criteria (attributes). More specifically, the items of the tool were verified as covering the entire set of clinical criteria (all columns contained at least one “1”). This result was achieved through the agreement of four specialists in the field of mood disorders selected on the basis of their expertise in the field of CBT and psychological assessment. More specifically, experts were asked to fill independently a Boolean matrix with the items in rows and the attributes in columns. Whenever an item, in their opinion, investigated a specific attribute, the corresponding cell in the matrix should have been filled with 1, otherwise with 0. Experts were not allowed to propose new items or new attributes. The Cohen's κ coefficient was computed for each pair of experts' matrices and resulted in an average value of 0.88 indicating a very good agreement among experts. The remaining disagreements were discussed and solved by means of a focus group.

Table 1 summarizes all of the sets of clinical criteria that we considered in constructing the MDE assessment tool.

**Table 1:** **The 23 clinical criteria for major depressive episode construct**.

**Attribute**	**Explanation**
A1	Depressed mood
A2	Diminished interest and pleasure
A3	Decreased interest in sex
A4	Increase or loss of weight
A5	Gain or loss of appetite
A6	Insomnia or hypersomnia
A7	Agitation
A8	Psychomotor retardation
A9	Fatigue or energy loss
A10	Feelings of worthlessness (or Beck's negative view of self)
A11	Feelings of guilt
A12	Diminished ability to think and concentrate
A13	Indecision
A14	Recurrent thoughts of death
A15	Suicidal ideation or attempted suicide
A16	Beck's negative view of the world
A17	Beck's negative expectation of the future
A18	Seligman's learned helplessness
A19	Irritability
A20	Apathy
A21	Health concern
A22	Somatic disorders
A23	More positive mood in the evening

First of all, MDE is described by the decomposition of the DSM-5 diagnostic criteria (American Psychiatric Association, 2013) for this disorder (it may be part of both MDD and BD); then, all of the described clinical criteria were taken into account: A1 (depressed mood), A2 (diminished interest and pleasure), A3 (decreased interest in sex), A4 (increase or loss of weight), A5 (gain or loss of appetite), A6 (insomnia or hypersomnia), A7 (agitation), A8 (psychomotor retardation), A9 (fatigue or energy loss), A10 (feelings of worthlessness), A11 (feelings of guilt), A12 (diminished ability to think and concentrate), A13 (indecision), A14 (recurrent thoughts of death), and A15 (suicidal ideation or attempted suicide).

Criterion A15 underlines the seriousness of “suicidal ideation” in MDE and is obviously the most severe symptom (Balázs et al., 2006; Batterham et al., 2015). It has been decided to separate this symptom by thoughts of death. According to several authors, suicide is indeed the third-highest cause of death in the population between 15 and 35 years old (Gunnell and Middleton, 2003; Baldessarini et al., 2006).

Furthermore, attributes A16, A17, and A18 are related to two theories of cognitive behavioral matrices, which led to the development of psychotherapeutic techniques that are now widely used: Beck's hopelessness theory and Seligman's helplessness theory.

- *Beck's hopelessness theory*. Beck's (1991; 2005) model contains persistent structural representations of human experience called schemes, which direct the identification, interpretation, classification, and evaluation of experiences. The schemes are structurally rigid, resistant, and absolute, and their content is a distorted representation of the experience. A negative view of oneself is a central feature of depression, and the real information about people's skills in various areas are distorted; also, patients with depression often have distorted evaluations of such events, as a result of the intervention of different processes called “cognitive distortions.”
  Beck found that beliefs and typical cognitive errors of depression are related to a “cognitive triad”:
  - A *negative view of oneself* in terms of personal value (“I am a loser”; “I am a failure”) and in terms of kindness and esteem of others (“nobody loves me”; “I'm not a person worthy of love”). The corresponding criterion has just been included in the DSM-5 criteria (A10).
  - A *negative view of the world* (“the world is a bad and unfortunate place”; “others take advantage of me”; “life is unfair toward me”); the corresponding criterion is A16.
  - A *negative expectation about the future* (“it will not change anything”; “I will always be a loser”); the corresponding criterion is A17.
- *Seligman's learned helplessness theory*. Seligman (1972), in an analogy with observations of dogs, suggested that among depressed men with depression, there is a learned conviction that they cannot do anything to face stressful life events and, as a consequence, they passively accept the outcome of those events (Alloy et al., 1984). This attitude tends to be generalized to new situations because of negative expectations, with people developing a sense of learned helplessness at the cognitive level. The criterion corresponding to this theory is A18.

On the basis of a careful literature review, other clinical criteria for MDE were taken into account because they are often part of depressive symptoms, even in the absence of comorbidity with other disorders of Axis I (Goodwin and Jamison, 2007). These symptoms are widely described in the literature, and they are potentially able to discriminate between different types of MDEs:

Criterion A19 refers to irritability (Henderson et al., 2013; Pedrelli et al., 2013); a person with depression can easily feel frustrated, and this frustration often results in outbursts of anger.

Criterion A20 refers to apathy (Mulin et al., 2011; Alexopoulos et al., 2013). Patients with depression often are characterized by decreased emotional reactions to situations and events in everyday life. Apathy is expressed in the form of indifference, physical inertia, or lack of reaction when facing situations that would normally arouse interest or emotion, as well as a reduction of purposeful behavior, a lack of initiative, and submission in one's daily choices.

Criterion A21 refers to health concerns (House, 1989; Magariños et al., 2002). It can take on the characteristics of real hypochondria in MDE, and the concerns may be related to somatization disorders.

Criterion A22 refers to somatic disorders (Goodwin and Jamison, 2007; Al Busaidi, 2010; Campo, 2012), which can be expressed through a myriad of symptoms in people with MDE, including neurovegetative disorders, stomach cramps, vomiting, difficulty of digestion, diarrhea, palpitations, hyperventilation, paresthesia, sweating, flushing, tremors, headaches, increased heart rate, an urgent need to urinate often, a feeling of heaviness in the limbs or in the head, and back or muscle pain.

Criterion A23 was inserted as the last in the list of clinical criteria for assessing MDE. The literature review and the presence of this attribute in the items of almost all MDE scales analyzed by Serra et al. (2015) demonstrate its important contribution to depression evaluation. In fact, individuals with depression usually feel better at the end of the day when they can go to sleep and do not have to face their daily problems anymore.

These last five symptoms are present in many rating scales of depression (Serra et al., 2015). These symptoms are useful to differentiate symptomatology; in addition to agitated depression and more inhibited depression, other authors have differentiated “anaclitic depression,” with feelings of loneliness and abandonment, from “introjective depression,” with feelings of failure and worthlessness (i.e., Blatt, 2004).

On the base of the criteria described above, 41 items have been constructed. Some of them contain a single diagnostic criterion; for example, the item “I feel helpless in the face of life events” contains A18—the learned helplessness of Seligman. Other items were constructed to include two or more diagnostic criteria; for example, the item “I feel nervous about this sadness I never abandon” contains three diagnostic criteria: A17—Beck's negative expectation of the future, A1—depressed mood, and A7—agitation. In line with the FPA methodology, the matrix was built to analyze all of the relationships among these items and diagnostic criteria. The whole set of clinical criteria (attributes) in Table 1 were investigated by the set of 41 items. This tool goes beyond the score of the patient and investigates the diagnostic features implicated by the answers that an individual has given (Spoto et al., 2013; Bottesi et al., 2015b; Serra et al., 2015). The investigation of diagnostic features is fundamental from a clinical point of view, since it allows for personalized diagnosis, which could have a role in treatment decisions and strategies, since the psychological and pharmacological treatment of agitated depression is different from that of inhibited depression. This new perspective is more deeply explored in the Results and Discussion sections.

### Participants

The research participants who were tested were divided into clinical and non-clinical groups.

The clinical group consisted of 38 subjects with MDE (who were diagnosed with MDD or bipolar disorder, or else with MDE during their first access in the day hospital) of the Neurosciences, Mental Health, and Sensory Organ (NESMOS) Department of La Sapienza University, Rome. In particular, the patients included in the study comprised eight individuals who were on their first access to the day hospital, four people who had a reactive MDE (caused by a stressful event or a death event), two who had an MDE with familiar genetics in mood disorders highlighted by their medical history, and two who suffered from unspecified MDE. Eleven patients were suffering from MDE within a major depressive disorder; one patient of this group had comorbid obsessive compulsive disorder (OCD), one patient had comorbid social anxiety disorder, and two others had comorbid eating disorder (anorexia nervosa). Nine patients were suffering from an MDE within bipolar disorder type 1; two of them had comorbid OCD, and two other patients had comorbid eating disorder (anorexia nervosa and bulimia). Finally, ten patients were suffering from MDE in bipolar disorder type 2; three of them had comorbid OCD, two of them had comorbid panic disorder, and one of them had comorbid social anxiety disorder. The exclusion criteria were mental retardation and psychotic traits to avoid problems in interpreting the responses to the QuEDS. Of the participants, 47% were male and the remaining 53% were female. A majority of the participants had a high school diploma, and their ages ranged between 21 and 69 years.

The non-clinical group consisted of 265 Italian individuals from different regions. The convenience non-clinical sample of the present research included individuals recruited in the area of the University of Padova (both students and non-students). The exclusion criteria in the non-clinical group involved all individuals suffering from MDE (e.g., those who were under pharmacological or psychotherapeutic treatment for depression). Among these participants, 70% were female. A majority of participants had a high school diploma, and their ages ranged between 19 and 56 years.

### Clinical tools

#### The qualitative—quantitative evaluation of depressive symptomatology (QuEDS)

The QuEDS tool contains 41 dichotomous items constructed on the basis of 23 clinical criteria of major depressive episodes from the DSM-5 and the literature. It is composed of 41 items; the maximum score is 41, and the minimum is 0. It is assumed that if a person responds positively to an item, then he/she has the symptoms (in terms of clinical criteria or attributes) included in this item.

#### Depression-anxiety-stress-scale 21 (DASS-21)

The DASS-21 is the short version of the self-report test designed to measure the three related negative emotional states of depression, anxiety, and stress (Henry and Crawford, 2005; Bottesi et al., 2015a). DASS-21 contains seven items for assessing depression seven items for assessing anxiety and seven items for assessing stress. The Depression scale evaluates dysphoria, hopelessness, devaluation of life, self-deprecation, a lack of interest/involvement, anhedonia, and inertia. The Anxiety scale assesses autonomic arousal, situational anxiety, and subjective experience of anxious affect. The Stress scale (items) is sensitive to levels of chronic non-specific arousal. It assesses difficulty in relaxing as well as being easily agitated, irritable, and impatient. The respondents are asked to use a 4-point Likert scale to indicate the severity and frequency of symptoms.

### Procedure and administration

All of the research participants completed informed consent and sociodemographic forms before answering the questionnaire items. No time limit was imposed to complete the questionnaires. All 265 subjects of the non-clinical group completed the QuEDS for major depressive episodes.

A subgroup of 113 individuals of the non-clinical group also answered the self-report measure DASS-21 to evaluate the convergent and divergent validity of the QuEDS. Moreover, 63 out of these 113 subjects compiled the QuEDS twice, after 1 month, to evaluate the temporal stability of the scale (test–retest).

The clinical group responded to the QuEDS, after being tested on their depressive symptomatology through a depression rating scale (SCID-I) and diagnosed as MDE patients by NESMOS Department's psychiatrists.

At clinic intake, participants provided written, informed consent for potential research analysis and anonymous reporting of clinical findings in aggregate form, in accord with Italian legal and ethical requirements. For this reason an ethics review process is not needed for these non-invasive clinical studies.

The study was conducted in accordance with the Declaration of Helsinki. All participants entered the study of their own free will and provided their informed consent before taking part. They were informed in detail about the aims of the study, the voluntary nature of their participation, and their right to withdraw from the study at any time and without being penalized in any way. Furthermore, participants were allowed to ask for restitution about their own score, providing authors with their own auto generated code, used during the administration phase.

### Data analysis

The whole sample (*N* = 303) was composed by a non-clinical subsample (*N* = 265) and a clinical subsample (*N* = 38), no missing data were observed. Different kinds of data analyses were conducted to test the validity and reliability of the QuEDS. Inferential analyses were conducted by means of the software R 3.3.0 (R Core Team, 2013), while confirmatory factor analysis (CFA) was conducted by means of the software LISREL 8.80 (Jöreskog and Sörbom, 1986, 1989, 1993; Jöreskog, 2006).

Known-groups validity was evaluated by means of both a comparison between the scores of non-clinical group (*N* = 265) and clinical group (*N* = 38), and referring to the classical ROC curves approach on the whole sample.

The data of non-clinical participants (*N* = 265) were used to fit a hierarchical model with three sub-factors and a higher-level factor (depression). This model was compared to other three possible factorial structures using the data of the entire non-clinical subsample (*N* = 265). Convergent validity was evaluated by computing the correlation between the QuEDS and the DASS-21 scores using the data of 113 participants of the non-clinical subsample. The reliability of the scale was tested both with respect to the internal consistency and to the test-retest. For the internal consistency the data of all non-clinical participants (*N* = 265) were used, while, for the test retest, were used 63 participants of the non-clinical subsample. Content validity has been evaluated by referring to the FPA methodology. Finally, again by means of the FPA, the capability of the tool to clinically discriminate patients has been tested and reported.

## Results

### Construct validity

The construct validity of the QuEDS was evaluated by investigating its factorial validity and convergent–divergent validity.

#### Factorial validity

As described above, a hierarchical model could be the factorial solution that is most likely to represent the structure of the investigated construct. Since the QuEDS was constructed both specifically for evaluating depressive symptoms and sensitively to evaluate thoughts (cognitive), somatic aspects (somatic), and emotions (affective) related to depression, authors are interested in testing, on the non-clinical sample—*N* = 265— (Osborne and Costello, 2004; Garson, 2008), a hierarchical factorial structure (Berrios et al., 2015; Roberts et al., 2015) with three sub-factors (i.e., cognitive, affective, and somatic), all linked to a second-order factor (i.e., depression). Furthermore, in order to compare the fit of this model to different theoretically plausible solutions, it was compared with three other different factorial models to the collected data: (a) a model with one latent construct which we called depression; (b) a model with two latent factors, which we called the somatic-affective factor and cognitive factor; and (c) a model with three factors: cognitive, somatic and affective factors.

In the proposed hierarchical model, the items were grouped into the sub-factors as follows:

- The *cognitive factor* includes items 5, 6, 9, 10, 14, 19, 20, 21, 24, 25, 27, 30, 32, 33, and 41. It comprises symptoms related to the distortions of thought systems and also to feelings of guilt, helplessness, worthlessness, hopelessness, and death.
- The *somatic factor* includes items 1, 2, 3, 4, 11, 13, 16, 22, 23, 26, 28, 31, 35, and 39. It comprises symptoms related to fatigue, sleep, appetite, psycho-motor retardation, and other somatic disorders that often involve MDEs.
- The *affective factor* includes items 7, 8, 12, 15, 17, 18, 29, 34, 36, 37, 38, and 40. It comprises the emotions that characterize different types of MDEs, including sadness (common in all subtypes of MDE), apathy, irritability, agitation, and various concerns.

Table 2 displays a comparison of the fit indexes for the four tested factorial structures. The table shows that the hierarchical model fits the data better than any of the three other models. All of the fit indexes for the hierarchical model (with no use of modification indexes) had adequate values. More specifically, the ratio between the Chi-square and the degrees of freedom, the RMSEA, the CFI, and the NNFI showed a good fit, while the NFI indicated an adequate model fit (Marsh et al., 2004). Furthermore, no significant double loadings were observed, nor correlation among error terms.

**Table 2:** **The fit indexes of the four tested models**.

**Fit index**	**Mono-factorial**	**Two-factor model**	**Three-factor model**	**Hierarchical model**
χ^2^/*df*	2.07	1.85	1.83	1.75
RMSEA	0.064	0.057	0.056	0.053
NFI	0.84	0.86	0.86	0.87
NNFI	0.91	0.93	0.93	0.94
CFI	0.91	0.93	0.93	0.94
SRMR	0.103	0.094	0.092	0.084
AIC	1,775	1,603	1,591	1,532

For the hierarchical model, all of the items' saturations on the respective factors were significant and ranged between 0.26 and 0.76 for the cognitive factor; between 0.32 and 0.71 for the somatic factor; and between 0.33 and 0.70 for the affective factor (Table 3).

**Table 3:** **Factor loadings of each of the 41 items of the QuEDS**.

	**Cognitive factor**	**Somatic factor**	**Affective factor**
Item 5	0.59	–	–
Item 6	0.37	–	–
Item 9	0.50	–	–
Item 10	0.42	–	–
Item 14	0.72	–	–
Item 19	0.32	–	–
Item 20	0.62	–	–
Item 21	0.37	–	–
Item 24	0.66	–	–
Item 25	0.64	–	–
Item 27	0.26	–	–
Item 30	0.51	–	–
Item 32	0.62	–	–
Item 33	0.26	–	–
Item 41	0.76	–	–
Item 1	–	0.32	–
Item 2	–	0.46	–
Item 3	–	0.59	–
Item 4	–	0.37	–
Item 11	–	0.34	–
Item 13	–	0.42	–
Item 16	–	0.61	–
Item 22	–	0.39	–
Item 23	–	0.36	–
Item 26	–	0.42	–
Item 28	–	0.71	–
Item 31	–	0.55	–
Item 35	–	0.54	–
Item 39	–	0.39	–
Item 7	–	–	0.36
Item 8	–	–	0.39
Item 12	–	–	0.49
Item 15	–	–	0.45
Item 17	–	–	0.45
Item 18	–	–	0.62
Item 29	–	–	0.38
Item 34	–	–	0.70
Item 36	–	–	0.46
Item 37	–	–	0.65
Item 38	–	–	0.53
Item 40	–	–	0.33
Depression	0.77	0.70	0.91

*No double loading were observed. In the last line of the table are displayed the strengths of the links between the first and second-order factors*.

The results support the selection of the hierarchical model by confirming that its underlying factorial structure has a higher-order factor accounting for the relationship among lower-order specific factors (Subica et al., 2014). In second-order models, it is necessary for the lower-order specific factors to be correlated among each other and with the higher-order factor (Schmid and Leiman, 1957). In this specific case, the links between the sub-factors and the higher-order factor were in the range of 0.70–0.91, once more supporting the selected model.

#### Convergent–divergent validity

The convergent validity of the QuEDS was verified by comparing its scores in the non-clinical sample with those of the DASS-21 (which, as described above, is constituted by three sub-scores for Depression, Anxiety, and Stress). The correlations among the 113 subjects' scores in the QuEDS and the three sub-scales of DASS-21 were all significant. More specifically, the correlation between the QuEDS and the Depression subscale of the DASS-21 was *r* = 0.72 (*p* < 0.05); the correlation between the QuEDS and the Anxiety subscale of the DASS-21 was *r* = 0.39 (*p* < 0.05); and the correlation between the QuEDS and the Stress subscale of the DASS-21 was *r* = 0.59 (*p* < 0.05). These results are not surprising, since the depression construct may have several features in common with the stress and anxiety constructs. However, it has to be stressed that the correlation between the QuEDS and the Depression subscale was significantly higher than the correlation between the QuEDS and the Anxiety subscale (*z* = 3.32, *p* < 0.001); on the contrary, a not significant difference was observed between the correlation of the QuEDS with the Depression subscale and the correlation of the QuEDS and the Stress subscale (*z* = 1.71, *n.s*.). These results are different from what Dobson (1985) found and indicate a good divergent validity of the QuEDS with respect to tools measuring different but correlated constructs.

The correlations among the sub-factors (cognitive-somatic-affective) of the QuEDS and the subscales of the DASS-21 have also been computed, and the results are displayed in Table 4. While the correlation between the Anxiety subscale of the DASS-21 and the factors of the QuEDS was systematically and significantly lower than the correlations between the QuEDS subscales and the Depression subscale of the DASS-21 (cognitive: *z* = 2.57, *p* < 0.01; somatic: *z* = 2.24, *p* < 0.05; affective: *z* = 2.03, *p* < 0.05), the situation was the opposite with respect to the Stress subscale. In fact, all the correlations between the three sub factors of the QuEDS and the Depression subscale of the DASS-21 were not significantly higher than their correlation with the Stress subscale (cognitive: *z* = 1.65, *n.s*., somatic: *z* = 1.23, *n.s*.; affective: *z* = −0.11, *n.s*.). Table 4 displays the 7 × 7 correlation matrix of the QuEDS total score, QuEDS subscales, and the three subscales from the DASS-21.

**Table 4:** **The correlation matrix of the three subscales from the DASS-21, the three subscales of the QuEDS, and the total score of the QuEDS**.

	**DASS-21 depression**	**DASS-21 anxiety**	**DASS-21 stress**	**QuEDS-cognitive**	**QuEDS-somatic**	**QuEDS-affective**	**QuEDS-TOT**
DASS-21 depression	–						
DASS-21 anxiety	0.37	–					
DASS-21 stress	0.44	0.50	–				
QuEDS-cognitive	0.64	0.37	0.48	–			
QuEDS-somatic	0.53	0.25	0.40	0.37	–		
QuEDS-affective	0.58	0.34	0.56	0.61	0.46	–	
QuEDS-TOT	0.72	0.39	0.59	0.80	0.78	0.84	–

### Known-groups criterion validity

The scores of 38 patients with MDE were compared with those of the 265 non-clinical subjects to test the known-groups validity of the QuEDS. The clinical group obtained an average score of 28.5 (*sd* = 6.5), while the non-clinical group obtained an average score of 6.5 (*sd* = 6). The difference between the two groups, tested using a *t*-test for independent samples, was significant (*t*_299_ = −20.20; *p* < 0.001) and supported the validity of the QuEDS. Furthermore, in order to test the ability of the QuEDS in separating the two groups, an analysis based on the ROC curves has been carried out. Results showed a very good value of the AUC statistic (confidence interval 0.97–0.99). Moreover, an optimal threshold score of 19 was determined that allowed for a specificity of 0.98 and a sensitivity of 0.94.

### Reliability

A reliability analysis, on the non-clinical sample, showed that the QuEDS scale has a very good internal consistency, Cronbach's α = 0.948. Even the alpha values relative to the three sub-factors were good: cognitive factor (α = 0.91; average item intercorrelation = 0.41), somatic factor (α = 0.86; average item intercorrelation = 0.31), affective factor (α = 0.82; average item intercorrelation = 0.41). Given such values and the number of items in the scale the alpha precision can be considered adequate (Cortina, 1993). Regarding the test–retest reliability of the QuEDS, the correlation among the scores of the 63 test subjects at Time 1 and Time 2 (after 1 month) was 0.74, which indicates good stability for the tool.

### Content validity

The QuEDS was created to answer the following question: Does it include the most common symptoms related to various types of MDEs? The FPA methodology was used to answer this question. A matrix was created with 41 items in the rows and 23 clinical criteria in the columns, called the “*clinical context*.” Thus, it was verified that each item would include one or more clinical criteria. Four specialists in the field of mood disorders carried out this analysis. Table 5 shows the content of the items and the set of attributes (symptoms) that each item investigates.

**Table 5:** **The items with their attributes**.

**Items**	**Clinical criteria**
1. I feel that I don't have the same energy to have sex	A3, A9
2. I often wake up in the middle of the night and I can't asleep again	A6, A7
3. I feel like that my thinking is slowing down	A8, A12
4. I have sleeping problems	A6
5. I am stressed by feeling of guilt	A11
6. I am think the world is cruel and unhappy	A16
7. I keep crying very easily	A1, A7, A19
8. I get irritated very easily	A19
9. I think my life is hell and I only deserve to feel bad	A1, A11, A16
10. I fell incapable to face life's events	A18
11. I suffer of somatic disorders (e.g. headache stomach ache)	A22
12. I have lost interest in the future which doesn't save anything good for me	A2, A17
13. I am less interest in sex	A3, A20
14. I feel incapable and totally useless	A10, A18
15. I see the same unhappiness I have now in the future	A1, A17
16. My desire to eat is not the same	A5
17. I often feel like crying, but I cannot do it	A1, A20
18. I cannot have any interest and pleasure in people and things that before I was interested in	A2, A20
19. I thought to kill my self	A14, A15
20. Sometimes I think it would be better if I were dead	A14
21. I am really worried about my health	A21
22. My weight has had significant changes	A4
23. I've visibly lost (or gained) weight	A4
24. I am afraid of about everything that it will happen to me because I am not able to do anything	A17, A18
25. I feel like I don't have any more power over my empty and sad life	A1, A16, A18
26. My appetite has changed	A5
27. To make choices is hard for me	A12, A13, A20
28. I feel I ‘m slowing down in my daily routines	A8, A9
29. I feel helpless and inhibited facing my incapacity to concentrate	A12, A20
30. I feel too much on the other people that it would be better if I killed myself	A10, A11, A14
31. I have not much energy and I feel tired	A9
32. I am disappointed of myself and the choices I made	A10, A11
33. I have problems in making decisions	A13
34. I feel sad	A1
35. My ability to think and memorize has been reduced	A8, A9, A12
36. I don't have any interest and desire in doing anything	A2
37. I am agitated of the idea that this sadness won't ever leave me	A1, A7, A17
38. I feel agitated	A7
39. I feel so tired and without any energy that I need help to wash myself and to get dressed	A8, A9, A18, A20
40. I am better in the evening more than in the morning	A23
41. I often feel like a loser	A10

Concerning content validity, the first key result is that the QuEDS was able to collect all of the information from 41 items, in terms of clinical criteria, to evaluate different types of MDEs. In addition, the items in the table above may include one or more clinical criterion. The literature and tests frequently used to assess depression highlight that none of the tests alone is able to investigate all clinical criteria, even those related to the DSM-5.

Furthermore, in formulating the items, we avoided methodological problems such as double phrases (“I'm depressed” or “I often want to cry”), fuzzy adverbs (“my life is pretty full”), or problems with content validity. In other words, each item of the QuEDS includes one or more clinical criteria described in Table 1; none of the items investigate other criteria or other symptoms that may be related to depression but are not part of the construct (e.g., items about anxiety, obsessions, etc.). In the matrix, this means that there were no empty rows (with all “0”). Also, there were no empty columns in the matrix; from a conceptual point of view, this means that the 41 items of the QuEDS investigate all 23 of the clinical criteria in Table 1.

By applying FPA, it became possible to conduct a content analysis even for the three sub-factor included in the model. For each factor, it has been possible to create a clinical context including all of its items and the subset of attributes investigated by the items of the sub-factor. The results of this procedure are displayed in Table 6.

**Table 6:** **The clinical contexts of QuEDS three factors**.

	**A1**	**A10**	**A11**	**A12**	**A13**	**A14**	**A15**	**A16**	**A17**	**A18**	**A21**
**COGNITIVE FACTOR**											
Item 5	0	0	1	0	0	0	0	0	0	0	0
Item 6	0	0	0	0	0	0	0	1	0	0	0
Item 9	1	0	1	0	0	0	0	1	0	0	0
Item 10	0	0	0	0	0	0	0	0	0	1	0
Item 14	0	1	0	0	0	0	0	0	0	1	0
Item 19	0	0	0	0	0	1	1	0	0	0	0
Item 20	0	0	0	0	0	1	0	0	0	0	0
Item 21	0	0	0	0	0	0	0	0	0	0	1
Item 24	0	0	0	0	0	0	0	0	1	1	0
Item 25	1	0	0	0	0	0	0	1	0	1	0
Item 27	0	0	0	1	1	0	0	0	0	0	0
Item 30	0	1	1	0	0	1	0	0	0	0	0
Item 32	0	1	1	0	0	0	0	0	0	0	0
Item 33	0	0	0	0	1	0	0	0	0	0	0
Item 41	0	1	0	0	0	0	0	0	0	0	0
	**A3**	**A4**	**A5**	**A6**	**A7**	**A8**	**A9**	**A12**	**A18**	**A20**	**A22**
**SOMATIC FACTOR**											
Item 1	1	0	0	0	0	0	1	0	0	0	0
Item 2	0	0	0	1	1	0	0	0	0	0	0
Item 3	0	0	0	0	0	1	0	1	0	0	0
Item 4	0	0	0	1	0	0	0	0	0	0	0
Item 11	0	0	0	0	0	0	0	0	0	0	1
Item 13	1	0	0	0	0	0	0	0	0	0	0
Item 16	0	0	1	0	0	0	0	0	0	0	0
Item 22	0	1	0	0	0	0	0	0	0	0	0
Item 23	0	1	0	0	0	0	0	0	0	0	0
Item 26	0	0	1	0	0	0	0	0	0	0	0
Item 28	0	0	0	0	0	1	1	0	0	0	0
Item 31	0	0	0	0	0	0	1	0	0	0	0
Item 35	0	0	0	0	0	1	1	1	0	0	0
Item 39	0	0	0	0	0	0	1	0	1	1	0
	**A1**	**A2**	**A7**	**A12**	**A17**	**A19**	**A20**	**A23**			
**AFFECTIVE FACTOR**											
Item 7	1	0	0	0	0	1	0	0			
Item 8	0	0	0	0	0	1	0	0			
Item 15	1	0	0	0	1	0	0	0			
Item 17	1	0	0	0	0	0	1	0			
Item 18	0	1	0	0	0	0	1	0			
Item 29	0	0	0	1	0	0	1	0			
Item 34	1	0	0	0	0	0	0	0			
Item 36	0	1	0	0	0	0	1	0			
Item 37	1	0	1	0	0	0	0	0			
Item 38	0	0	1	0	0	0	0	0			
Item 40	0	0	0	0	0	0	0	1			

Each row is an item, while each column is a clinical criteria either investigated or not by the item. Every time an item investigates a specific criterion, the corresponding cell will contain “1,” (otherwise “0).”

It is noteworthy how the sets of attributes investigated by each factor are different (Table 7). More specifically, only the cognitive factor investigates feelings of worthlessness and guilt, indecision, recurrent thoughts of death and suicidal ideation, Beck's negative view of the world, and finally health concerns; in fact, all of these symptoms are related to thoughts. The somatic factor alone includes decreased interest in sex, increased or loss of weight, gain or loss of appetite, insomnia or hypersomnia, psychomotor retardation, fatigue or energy loss, and somatic disorders; all of these manifestations are physical dysfunctions. Instead, the affective factor alone comprises diminished interest and pleasure, irritability, and a more positive mood in the evening. Finally, some symptoms are investigated by two sub-factors, and one of them is investigated by all of the sub-factors.

**Table 7:** **The factors that investigate each single attribute of the clinical context**.

**Factors**	**Attributes**
All three factors	A12
Cognitive and affective	A1, A17
Cognitive and somatic	A18
Somatic and affective	A7, A20
Cognitive	A10, A11, A13, A14, A15, A16, A21
Somatic	A3, A4, A5, A6, A8, A9, A22
Affective	A2, A19, A23

The following table briefly describes the links between the sub-factors and the attributes (symptoms) that they share.

The strong innovation of FPA comes from the construction of the matrix, which allows identification of the actual existing relations between the items and clinical criteria as well as among the items, in terms of the clinical symptoms they endorse. Analysis of these relationships allows clinicians to go beyond the score, acquire qualitative information on the individual, and understand and analyze the patient's symptomatic areas in an adaptive way. A descriptive example about how the FPA could integrate the quantitative information collected through the questionnaire is presented below.

#### Beyond the numeric score: the “clinical state” of the patient

As stated before, the new QuEDS allows clinicians to go beyond the numeric score and focus their analysis on the symptoms that patients experience or about which they complain. In fact, the QuEDS takes into account all of the positive responses of the subject, which are closely linked to the symptoms through the FPA (MDE clinical criteria). Clinicians will no longer be bound to the patient's score but will be interested in the patient's clinical state, which is the set of items to which the patient responded positively, along with the set of symptoms investigated by those items. Such information is already present in the items, but it is hidden by a classical testing methodology that considers the questionnaire score to be the most relevant output used by the clinicians.

It may be useful to introduce a practical example from the patients of this study; two of the 38 patients in the clinical group mentioned above were chosen who obtained the same score on the QuEDS: 31. This means that both answered “yes” to 31 items out of the 41 total items. This score is clearly high, and the patients—who had already been diagnosed with MDE—were confirmed to have a depressive symptomatology with this scoring. However, the two patients did not have exactly the same disorder. Patient SC was suffering from a reactive MDE (subsequent to a stressful life event), while patient FG was suffering from MDE inside bipolar disorder type 1 (see DSM-5). Many authors (e.g., Koukopoulos and Koukopoulos, 1999; Maj et al., 2003) have shown that MDEs in bipolar disorder often occur with more agitated features. According to the classical methodology, the questionnaire's output is the same for both patients. In agreement with several other authors (Wright and Feinstein, 1982; Gibbons et al., 1985; Fava et al., 2004; Bottesi et al., 2015b; Serra et al., 2015), in this study, it is assumed that if the two patients had the same score, this did not mean that they had equal symptomatology (they may have answered affirmatively to the same number of items but not to the same items, and the whole symptomatology may be more serious in one of them). Unlike in the usual methodology, qualitative information on the two patients' symptoms was collected through their clinical state.

Patient SC responded affirmatively to items 1, 3, 5, 6, 10, 12, 13, 14, 15, 16, 17, 18, 20, 22, 23, 24, 25, 26, 27, 28, 29, 30, 31, 32, 33, 34, 35, 36, 39, 40, and 41. Consequently, his clinical state contained the following symptoms (attributes) in terms of clinical criteria: A1, A2, A3, A4, A5, A8, A9, A10, A11, A12, A13, A14, A16, A17, A18, A20, and A23. Patient FG responded affirmatively to items 2, 4, 5, 6, 7, 8, 9, 10, 11, 12, 13, 14, 15, 16, 18, 19, 20, 21, 24, 25, 26, 27, 29, 32, 33, 34, 36, 37, 38, 40, and 41. Accordingly his clinical state contained the following symptoms (attributes) in terms of clinical criteria: A1, A2, A3, A5, A6, A7, A10, A11, A12, A13, A14, A15, A16, A17, A18, A19, A20, A21, A22, A23 (to see which criteria the items are related to, see Tables 1, 5).

The listed attributes of both patients comprise the items they answered positively. As can be seen, the two patients had two different clinical states. Specifically, they shared a large number of attributes (namely, A1, A2, A3, A5, A10, A11, A12, A13, A14, A16, A17, A18, A20, and A23). This fact indicated that many of the general characteristics of the disorder presented by both patients were the same. Nevertheless, each patient presented some specific characteristics that the other did not share. These characteristics discriminate between the two clinical conditions and are crucial for effective treatment. More specifically, patient SC had the following additional symptoms: A4 “increase or loss of weight,” A8 “psychomotor retardation,” and A9 “fatigue or energy loss.” On the other hand, patient FG, in addition to the shared symptoms, presented the following attributes: A6 “insomnia,” A7 “agitation,” A15 “suicidal ideation,” A19 “irritability,” A21” health concerns,” and A22 “somatic disorders.”

Furthermore, the two patients' responses to the items of the three sub-dimensions of the QuEDS (cognitive, somatic, and affective) were considered. Patient SC responded positively to 12 out of 15 items of the cognitive factor, 11 out of 14 items of the somatic factor, and 7 out of 12 items of the emotional factor; in contrast, FG responded positively to 14 out of 15 items of the cognitive factor, 6 out of 14 items of the somatic factor, and 11 out of 12 items of the affective factor. It has to be stressed, however, that the mere score to the subscales of the QuEDS, even if useful to help clinicians in preliminarily understand the situation of patients, is not sufficient to clearly differentiate the specific kind of depression characterizing the two patients. In fact, neither a high score in the affective factor implies an agitated depression, nor a high score in the somatic factor implies the presence of an inhibited depression. Such characterizations can be, on the contrary, easily deduced by the clinical states provided through the FPA approach. Furthermore, even equal scores to the same subscale may be due to different clinical states. For instance, considering the Affective factor, it can be seen that it contains items conveying rather different clinical information. In other words, the same score on the same subscale can be attained even if the collection of attributes presented is slightly different: consider the response patterns *A* = 7, 8, 15, 34, 37, 38, 40; and *B* = 12, 15, 17, 18, 29, 34, 36. They equally score 7. Nevertheless, the attributes presented by a patient who endorses the response pattern A are A1, A7, A17, A19, and A23; while those presented by a patient with response pattern B are A1, A2, A12, A17, and A20. Both patients present depressive mood (A1) and Beck's negative expectation of the future (A17), but A is more concerned with irritability and agitation, while B is characterized by apathy.

This analysis allows a better classification of the individual symptoms' case, which therefore allows for planning different pharmacological and psychological treatments for the two patients. The qualitative differences in symptoms between the two patients are highly relevant for a correct diagnosis and for future psychological and pharmacological treatment. It is noteworthy to observe that doctors sometimes prescribe antidepressants without carefully analyzing the individual's depressive symptoms, yet these drugs can be very dangerous and can increase the risk of suicide in people with agitated depression (Balázs et al., 2006; Baldessarini et al., 2006).

Considering a cognitive behavioral and pharmacological approach, both patients may need some techniques to identify and correct their negative automatic thoughts and cognitive distortions. However, SC showed inhibited symptoms such as psychomotor retardation and a lack of energy, which might be linked to reduced activity as well as overlooking one's duties and daily responsibilities because of thoughts like “I do not have the strength” and “I will not make it ever.” A goal of psychological treatment may be to eliminate the guilt for these disabilities and to encourage the patient to perform one small task at a time, while an antidepressant may be useful as a drug treatment. On the other hand, patient FG responded to symptoms of restlessness, including insomnia, irritability, health concerns, agitation, and in particular, suicidal ideation. FG's psychotherapeutic intervention may be more focused on body relaxation and advice on the conduct of daily life (e.g., avoid excessive working stress and follow a regular rhythm of life), while the drug therapy may be in line with bipolar disorder and include mood stabilizers and sedative drugs to reduce agitated depressive episodes.

Moreover, the proposed methodology even provides information to clinicians about the possible symptoms of a person who does not have MDEs and who belongs to the non-clinical group. To illustrate, MT obtained a score of six to the questionnaire (the mean of the non-clinical group) after responding affirmatively to items I1, I11, I13, I27, I31, and I33. His clinical state was A3, A9, A13, A20, and A22. Even if he did not show a depressive symptomatology, dysfunctions related to his sexual desire, his energy, and his indecision emerged from his clinical state; also, he has somatic complaints. A usual questionnaire only provides a quantitative score (6), which only means that MT is not suffering from MDEs. This information could underline symptoms in common with some other psychological disorder and could show alarming manifestations, which occur in a “broad spectrum” evaluation, in which the clinician understands some crucial symptoms of the subject and then explores them with more specific and targeted tests.

Therefore, the output of the proposed tool (QuEDS) is the patient's clinical state; it is no longer the score. From a clinical point of view, a qualitative self-report tool overcomes the cutoff limit, which can be helpful just to have an idea about the test score, but it cannot be mistaken for a correct estimate of a person's symptomatology.

## Discussion

According to the DSM-5, there are different types of MDE, and depressive symptoms may have different features, depending on the individual and his/her particular disorder. The present paper was aimed at introducing a new assessment tool capable of account for the differences among the clinical symptomatology of patients that are not evaluable using traditional test scores alone. This task was carried out by using FPA as the theoretical framework in constructing the tool. Concerning the different clinical features, some specific illustrations showed how the tool can be used to more deeply investigate the clinical state of different patients.

Statistical results confirmed the goodness of the proposed tool, in terms of both validity and reliability. The high internal consistency of both the subscales and the whole scale indicates how the items are coherent in exploring the construct. With respect to the test-retest reliability, the correlation shows a good stability of the measure. It has to be noted that, given the 1 month time interval selected for the retest, this reliability estimate may not be replicable with participants whose depressive symptoms are more truly episodic.

Concerning the factor structure, the hierarchical model explains best the observed data. As shown in the results MDE can be explained both by the general “depression” factor and by the three sub-factors (cognitive-somatic and affective), and a patient may have more somatic symptoms, or more cognitive/affective symptoms depending on the features of his illness. Also this aspect may become important for the treatment of the individual case.

The results of the divergent/convergent validity on the one hand showed the difference between the correlation of anxiety subscale (A) of the DASS and QuEDS and the correlation between the depression subscale (D) of the DASS and QuEDS, highlighting the convergent validity between the D-scale and QuEDS; on the other hand, the correlation between the subscale stress (S) of the DASS and QuEDS showed the presence of many shared clinical features between the two constructs. It is noteworthy that especially the affective and somatic sub-factors of the QuEDS have a high correlation with the scale S. This result may seem unusual at first, but as the literature suggests (Hewitt and Flett, 1993; Dumont and Provost, 1999; Tafet et al., 2001) stress and depression have many symptoms in common, in particular the people vulnerable to mood disorders are more sensitive to stress (Bidzi, 1984). Furthermore, the Stress scale of DASS is sensitive to levels of chronic non-specific arousal. It assesses difficulty relaxing, nervous arousal, and being easily upset/agitated, irritable/over-reactive and impatient; all these symptoms are highly correlated to the affective dimensions, as our results suggested.

Concerning the content validity, from this new perspective, the relations among the 41 items and the 23 clinical criteria play a crucial role. The matrix shows that of the all clinical criteria are investigated by at least one item. This result is very important because it means that the presence or absence of the 23 symptoms selected for describing the MDE can be detected using the QuEDS. Having information on all of these symptoms makes it possible to compare the observed responses of the subject according to the clinical symptoms he/she demonstrated up to the present. This fact, for instance, allows for determining whether an individual has more inhibited symptoms, more agitated ones, or both. Indeed, the set of items to which the individual responds includes a well-defined series of clinical criteria, which are useful for a first psycho-diagnostic examination. Thus, the output of the QuEDS is no longer crucially related to some sort of cutoff (or score) that shows whether the person could be classified as suffering from the disease or not. On the contrary, it consists of qualitative and quantitative information about the patient's clinical state. Such output provides the potential capability to go beyond the scores and investigate the configuration of the patients' symptoms to differentiate people who received the same score on the test but have different symptoms (by responding affirmatively to the same number of items but not to the same items) and considering when the whole symptomatology is more serious.

Moreover, unlike many self-report measures in use to assess MDEs, the QuEDS deeply investigates the symptoms related to agitated depression, including irritability, insomnia, crying spells, somatic disorders, and agitation. The investigation the symptoms related to agitated depression is quite important because people with agitated depression need completely different pharmacological and psychological treatments from those with other types of MDE; for example, antidepressants can increase the psychomotor agitation and the risk of suicide in people with agitated depression (Baldessarini et al., 2006; Balázs et al., 2006).

Another important result obtained by the QuEDS is the possible future application of an adaptive logic based on the matrix in Table 6. The reasoning is the same as the semi-structured interviews (Ferentinos et al., 2011). In a future direction, the QuEDS will not follow a predetermined sequence of items, since the items' order will be presented by a computerized routing on the basis of the previously collected answers. Given this procedure, it will probably be unnecessary to administer the whole test to the subject, but only the items related to the subject's replies, deepening the symptomatic areas while not investigating the areas in which the subject does not present those specific symptoms. Since we divided the matrix of 41 items into three sub-matrices related to the three sub-factors—cognitive, somatic, and affective (Table 6). This could be useful for the future implementation of the three algorithms of the QuEDS to respond adaptively and individually to the test (the mathematical steps are beyond the aims of this study; Donadello et al., 2016).

To summarize, the purpose of this study was to create an innovative tool that takes into account the weaknesses of the tests being used and their real potential in the assessment phase. It is considered that a psychological assessment cannot be conducted using only self-report tools; rather, effective and efficient tools to support the diagnosis and treatment are an integral part of the assessment. As a consequence, our proposed QuEDS could be a useful contribution for many reasons: first, for the broad spectrum of clinical criteria investigated by the test; second, for the importance given to qualitative information about the symptoms (through the patient's clinical state) and not only to the score; third, for the relevance attributed to the differences in symptoms and especially in their severity; fourth, for the possible future application of an adaptive logic; and finally, for its appreciable validity and reliability results.

## Author contributions

FS, AS, MG, and GV: project design; FS and AS: introduction and discussion writing; FS: participants recruitment and testing; AS, FS, and GV: data analysis; FS, AS, and MG: methods and results writing; GV: project supervision.

### Conflict of interest statement

The authors declare that the research was conducted in the absence of any commercial or financial relationships that could be construed as a potential conflict of interest.
